# Total Flavone of *Abelmoschus manihot* Ameliorates TNBS-Induced Colonic Fibrosis by Regulating Th17/Treg Balance and Reducing Extracellular Matrix

**DOI:** 10.3389/fphar.2021.769793

**Published:** 2021-12-23

**Authors:** Lichao Qiao, Lei Fang, Junyi Zhu, Yu Xiang, Haixia Xu, Xueliang Sun, Hongjin Chen, Bolin Yang

**Affiliations:** ^1^ Department of Colorectal Surgery, Jiangsu Province Hospital of Chinese Medicine, Affiliated Hospital of Nanjing University of Chinese Medicine, Nanjing, China; ^2^ First Clinical Medical College, Nanjing University of Chinese Medicine, Nanjing, China; ^3^ Department of Colorectal Surgery, Suzhou TCM Hospital Affiliated to Nanjing University of Chinese Medicine, Suzhou, China

**Keywords:** *Abelmoschus manihot*, Crohn’s disease, anti-fibrosis, Th17/Treg cells, extracellular matrix

## Abstract

**Background and Aims:** Surgery remains the major available strategy in inflammatory bowel disease (IBD) fibrotic strictures because no available drugs have sufficient prevention and treatment in this complication. This study aimed to evaluate the efficacy of the total flavone of *Abelmoschus manihot* L. Medic (TFA) on the development of colonic fibrosis in mice and its possible mechanism.

**Methods:** The 2,4,6-trinitrobenzene sulfonic acid (TNBS)-induced chronic colonic inflammation-associated fibrosis mice were used to evaluate anti-fibrosis of TFA using macroscopic, histological, immunohistochemical analyses, ELISA, Masson staining, Verhoeff’s von Gieson staining, transcription-quantitative polymerase chain reaction, and immunoblot analysis.

**Results:** Oral administration of TFA attenuated body weight loss, reduced colon length shortening, lowered the morphological damage index score, and notably ameliorated the inflammatory response. TFA downregulated proinflammatory cytokines IL-6, IL-17, TNF-α, IFN-γ productions, and increased the levels of anti-inflammatory cytokine IL-10 and TGF-β. The histological severity of the colonic fibrosis was also notably improved by the TFA treatment and associated with a significant reduction in the colonic expression of col1a2, col3a2, and hydroxyproline. TFA inhibits α-SMA, TGF-β, vimentin, TIMP-1 expression, increasing MMPs, thereby inhibiting activated intestinal mesenchymal cells and extracellular matrix (ECM) deposition.

**Conclusion:** Together, we herein provide the evidence to support that TFA may restore the imbalance of Th17/Treg and decrease the generation of ECM. This may be a potential mechanism by which TFA protects the intestine under inflammatory conditions and acts as a therapeutic agent for the treatment of intestinal fibrosis in Crohn’s disease.

## Introduction

Crohn’s disease (CD) is a chronic inflammatory bowel disease that affects any part of the gastrointestinal tract, and its prevalence has increased annually worldwide. CD manifests with abdominal pain, chronic diarrhea, and weight loss in the clinic. With progressive bowel damage, complications including strictures and/or penetrating lesions gradually arise (Freeman, 2003). CD is presumed to be an idiopathic chronic autoimmune disorder, characterized by T-cell dysfunction. The imbalance of Th17/Treg cells is at the heart of its pathogenesis (Brand, 2009).

Population-based cohort studies showed that up to 40% of patients with CD will develop intestinal fibrosis, which involves all intestinal layers and lead to strictures and even intestinal obstruction (Peyrin-Biroulet et al., 2010; Cosnes et al., 2011). Fibrogenesis is an adaptive response to the onset of inflammation and facilitates tissue repair, whereas fibrosis is an exaggerated response to chronic inflammation and leads to excessive scarring characterized by profound production and deposition of extracellular matrix (ECM). The ECM is formed primarily of collagen subtypes I, III, and V and proliferation of mesenchymal cells mainly fibroblasts, myofibroblasts, as well as smooth muscle cells, leading to stiffness and remodeling (Graham et al., 1988). The deposited ECM is usually degraded by matrix metalloproteinases (MMPs), which can be inhibited by tissue inhibitors of metalloproteinases (TIMPs). The fibrosis will occur when ECM production is increased and surpasses degradation (Rieder et al., 2017).

Intestinal fibrosis is tightly linked to chronic inflammation, but a sufficient body of evidence suggests that once fibrosis is initiated, it seems to progress independently from inflammation (Wu and Chakravarti, 2007; Rieder et al., 2017). Therefore, despite a great advance in pharmacological treatment, especially new biologic therapies targeting different immune pathways have been introduced over recent years, and no effective anti-fibrotic agents have been released to date. In contrast to recent advances in anti-inflammatory treatment, little therapeutic progress has been made regarding anti-fibrotic treatment. Endoscopic interventions or surgery such as stricture plasty or bowel segment resection remain as major therapeutic approaches for patients with symptomatic strictures, especially CD (Rieder et al., 2013). Overall, 70% of CD patients with fibrotic stenosis require partial bowel resection within 10 years of disease progression (Zorzi et al., 2015). Fibrosis-related complications are associated with a high socioeconomic burden due to frequent hospitalizations and surgeries (Yu et al., 2008; Rao et al., 2017).

There is an urgent and unmet need to expedite the development of anti-fibrotic drugs for IBD. Antifibrotics developed from natural products for natural medicines may cause hypotoxicity and are time saving. Total flavone of *Abelmoschus manihot* L. Medic (TFA) is the main flavonoid compound extracted from *Abelmoschus manihot*, which has been traditionally used in Chinese medicine to treat infectious diseases, malignant sores, or cellulitis, due to its anti-inflammatory and regulating immune function. Pharmacological and clinical studies have demonstrated that the extract of its flowers possessed various biological activities, including analgesic, anti-inflammatory, antioxidant, renal protection, gastric protection, etc. (Luan et al., 2020). Previous studies have reported that TFA can improve renal tissue fibrosis and reverse renal fibrosis in model mice (Chen et al., 2012). Our findings indicated that TFA could suppress the inflammatory response in mice with TNBS-induced colitis *via* inhibition of the NF-κB and MAPK signaling pathways (Zhang D. et al., 2019a). *In vitro* experiments have demonstrated that TFA inhibiting TGF-β induced epithelial-to-mesenchymal transition (EMT) in IEC-6 cells, which was typically considered to be a pivotal step in fibrosis (Yang et al., 2018). We also have demonstrated the anti-fibrotic actions of TFA in ameliorating TNBS-induced colitis fibrosis (Zhang et al., 2017), whereas, the specific mechanisms remain to be elucidated. This study was undertaken to validate the protection effect of TFA in an animal model of intestinal fibrosis and to further explore the possible mechanism of action.

## Materials and Methods

### Preparation of Total Flavone of *Abelmoschus manihot* L. Medic

*Abelmoschus manihot* was collected from Jiangyan of Jiangsu Province, China. TFA was extracted from the flowers of *Abelmoschus manihot* by the Department of Chinese Materia Medica, Nanjing University of Chinese Medicine, Nanjing, China. Powdered *Abelmoschus manihot* flowers were immersed in 75% ethanol for 1 h. The mixture was refluxed for 1 h at 90°C and filtered by analytical filter paper, and evaporated by rotary evaporation under vacuum at 60°C. For animal experiments, TFA was dissolved in sterile water.

### Animals

Sixty male BALB/C mice (6–8 weeks, 16–20 g) were purchased from the Animal Experimental Center of Nanjing Medical University (Nanjing, China). All mice were housed in plastic cages on a 12-h light/12-h dark cycle under controlled temperature (22 ± 2°C) and humidity (50 ± 10%), with *ad libitum* access to food and water. All animal welfare and experimental procedures followed the National Institute of Health (United States) guidelines, and were approved by the Animal Ethics Committee of Affiliated Hospital of the Nanjing University of Chinese Medicine.

### Induction and drug administration of chronic colitis

Chronic colonic inflammation-associated fibrosis was induced as reported previously (Tomasello et al., 2015). The mice were randomly divided into three groups: listed as 45% ethanol group of 12 mice, saline group of 12 mice, TNBS model group of 36 mice. Briefly, after being deprived of food overnight and lightly anesthetized with ether, 0.5 ml of 45% ethanol (sham-1), saline (sham-2), or TNBS (50 mg/kg) dissolved in 0.5 ml of 45% ethanol were instilled into the colon (3.5 cm proximal to the anus) through a flexible catheter carefully, and the animal was held in the Trendelenburg position for 1 min to ensure contact with the intestinal mucosa. The experiment was repeated weekly for 4 weeks. Subsequently, the mice with TNBS-induced colitis were randomly assigned to three treatments (12 mice/group), model group, TFA in the modeling group (TFA treatment was started at the beginning of TNBS induction), and TFA after the modeling group (TFA treatment starts 1 week after the end of TNBS induction). In the preliminary study, Masson staining showed that the fibrosis score and the percentage of collagen area decreased more significantly in the 250 mg/kg than in the 125 and 500 mg/kg groups, and therefore, this dose was used throughout the whole experiments (Zhang et al., 2017). TFA (250 mg/kg, dissolved in 0.5 ml of sterile water) was administered once daily *via* intragastric instillation every day for 4 weeks. Groups of sham-1, sham-2, and model received the sterile water by the same route.

Blood samples were taken by intracardiac puncture of mice under anesthesia before killing. Then the mice were sacrificed by neck dislocation, and the colonic samples were collected. All tissues were subdivided for RNA and protein isolation and histological analysis.

### Assessment of Colonic Damage

Bodyweight, stool features, and fecal occult blood of the mice were monitored throughout the experiment on alternate days. The disease activity index (DAI) was evaluated according to previously established criteria (Tomasello et al., 2015). The length and weight of colons were determined. Colon gross morphological damage index (CMDI) score was calculated according to previously established criteria (Wang et al., 2017). Scores of two gross morphology values were averaged for statistical analysis.

### Histopathological Examination

The colonic samples were fixed in 10% formalin, and paraffin embedded. Then the 5 μm thickness serial sections were subjected to hematoxylin/eosin (H&E) staining. Finally, the slides were examined under a light microscope (with ×100 magnification) to observe the morphological changes and recorded (Nikon 80i). Ten random microscopic fields from each tissue section were digitally captured under fixed microscope illumination settings. At least six colonic tissues were selected and analyzed for each animal.

### Masson Staining and Verhoeff’s van Gieson Staining

The paraffin blocks of colonic tissue were cut in 5 μm thickness, and sections were subjected to Masson trichrome staining following the manual. Photographs were taken by light microscope (Nikon 80i). Under the low-power microscope, the collagen fibers were stained blue, and the cytoplasm and muscle fibers were stained red. Then the percentage of the collagen fiber as a fraction of the overall area was calculated.

To visualize the collagen deposition and fibrosis in the colon, we used Verhoeff’s van Gieson staining. Colonic tissue sections were stained in Verhoeff’s staining solution for 30 min. The specimens were differentiated with 2% ferric chloride (Sigma-Aldrich) for 1 min and rinsed briefly in running tap water; the color changes were observed under a microscope to control the degree of dyeing. In the next steps, von Gieson solution (saturated picric acid, 1% acid fuchsine, and distilled water) was applied for 3-min staining. Finally, the specimens were dehydrated in graded alcohols and xylene. In the resulting slides, elastic fibers were black, collagen red, and background yellow. Finally, the percentage of the collagen fiber as a fraction of the overall area was calculated.

### Immunohistochemical Analysis

Immunohistochemical analysis was performed on paraffin-embedded colonic tissue sections (5 μm). The antibodies were used as follows: α-smooth muscle actin, TGF-β, Col1a2, Co13a2, and vimentin (Abcam, Cambridge, MA, USA). The sections were treated with 3% hydrogen peroxide in methanol to quench the endogenous peroxidase activity, followed by incubation with 1% BSA to block nonspecific binding. Then the primary antibody was incubated for 12 h at 4°C, and the second antibody was incubated for 1 h at room temperature. After staining of HRP deposits with diaminobenzidine (DAB) solution, the specimens were observed by a light microscope (Nikon 80i). For quantitative analysis of the results of immunohistochemistry, the average optical density was calculated as IOD/total area.

### Enzyme-Linked Immunoassay

Serum and colonic tissue levels of IL-6, IL-10, IL-17, TNF-α, IFN-γ, TGF-β, IGF-1, and hydroxyproline were determined using ELISA kits (Abcam, Cambridge, MA, USA), according to the instructions of the manufacturer.

### RNA Isolation and Quantitative Real-Time-PCR

TRIzol Reagent (Generay, China) was used for the extraction of total RNA from colonic tissue specimens. Complementary DNA was synthesized from 1 μg of total RNA using reverse transcriptase (Takara Bio Inc., Shiga, Japan). Gene expression was quantified using SuperReal PreMix Plus (with SYBR Green I) (Tiangen Biotech, Beijing, China) in a CFX Connect Real-Time PCR Detection System. The mRNA gene expression levels were normalized to GAPDH gene expression levels. The real-time PCR reactions were performed in triplicate.

### Western Blotting

The total protein was extracted from the colonic tissue using a lysis buffer. The protein concentration of the supernatant was determined using a BCA protein assay kit (Vazyme, USA). Samples (25 µg of protein) were separated on 10% SDS-PAGE gels and transferred to PVDF membranes and incubated with skim milk (5%) for 1 h at room temperature and then with primary antibodies overnight at 4°C. The primary antibodies included those that were selectively recognized: TGF-β, Col1a2, Col3a2, α-SMA (Abcam, Cambridge, MA, USA), followed by incubation with corresponding secondary antibodies. The signals were visualized using an ECL detection reagent (Yeasen Biotechnology, Shanghai, China), and the results were measured using ImageJ software 1.48 (National Institutes of Health).

### Statistical Analysis

Data analyses were performed using the R statistics package (R Statistical Software, version 3.3.3). The experimental data were presented as the mean ± standard deviation. Differences between experimental groups were assessed by one-way analysis of variance (ANOVA), followed by a least significant difference test, which was used to make comparisons among the groups. A value of *p* < 0.05 was considered statistically significant. In all figures, “*” represents *p* < 0.05, **p < 0.01, and ***p < 0.001 compared with the sham-1 and sham-2 groups; ^#^ *p* < 0.05, ^##^ *p* < 0.01, ^###^ *p* < 0.001 compared with the model group.

## Results

### Total Flavone of *Abelmoschus manihot* L. Medic is Protective Against Colitis Induced by 2,4,6-Trinitrobenzene Sulfonic Acid

**FIGURE 1:**
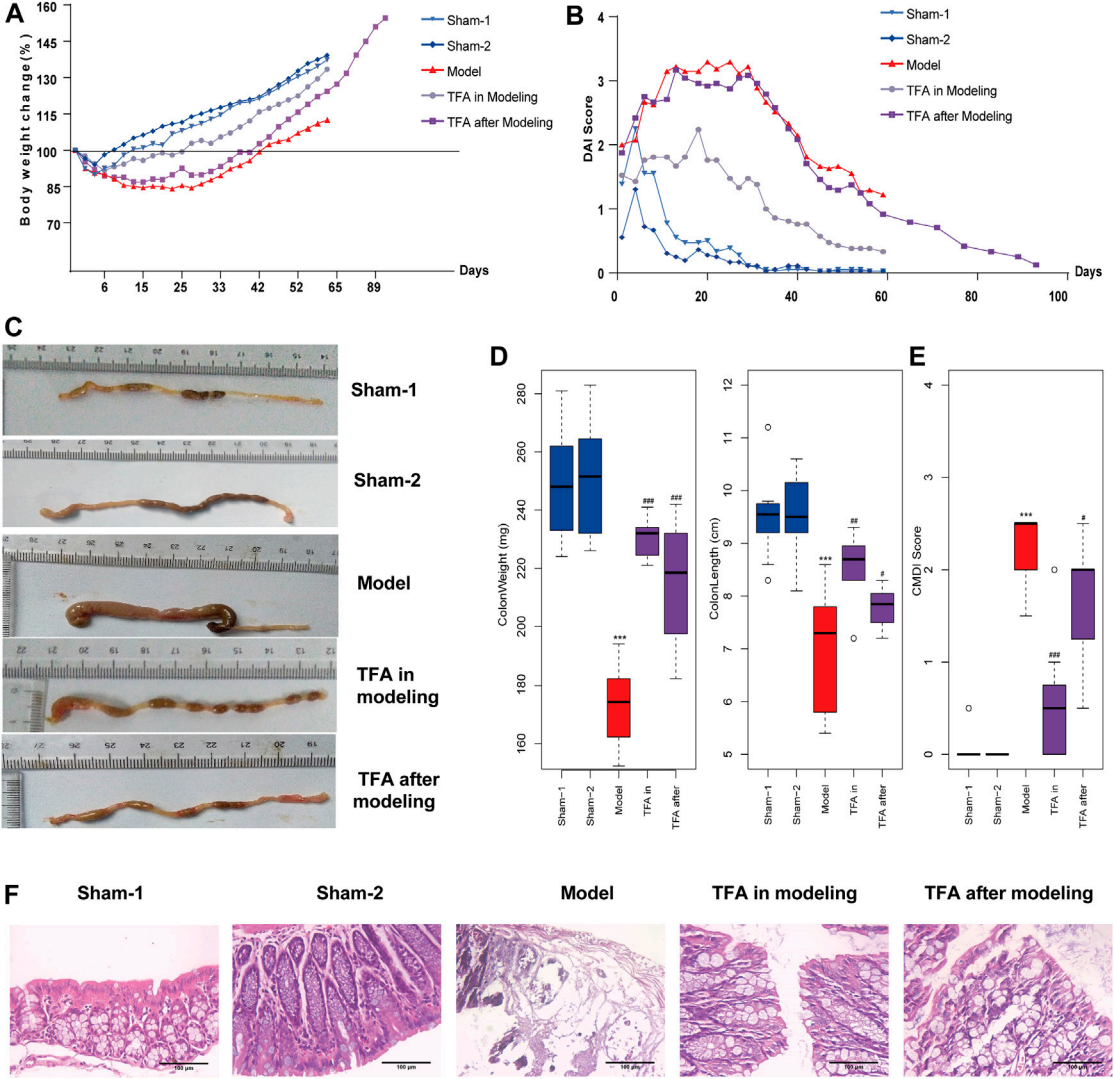
Total flavone of *Abelmoschus manihot* L. Medic (TFA) attenuates colitis in the 2,4,6-trinitrobenzene sulfonic acid (TNBS)‐induced colitis in mice. **(A)** Bodyweight change. **(B)** Disease activity index. **(C)** After sacrificing, colon damage was evaluated by colon length. **(D)** Length and weight of colon (from the appendix to the anus). **(E)** Colon gross morphological damage index (CMDI) score of each group. **(F)** Histological appearance of mice colonic mucosa (H and E, magnification ×100). Data are shown as the mean ± SEM. ***p < 0.001 compared with sham-1 and sham-2 groups, ^#^ *p* < 0.05, ^##^ *p* < 0.01, ^###^ *p* < 0.001 compared with the model group.

In this study, the TNBS-induced chronic colitis mice model was employed to evaluate the effect of TFA. As expected, a marked progressive weight loss and DAI score increase was observed in TNBS-induced mice compared with the sham-1, sham-2 groups, while treatment with TFA in modeling or after modeling showed significantly reversed body weight loss (Figure 1A) and DAI score (Figure 1B). Moreover, we found that the colon length and weight in the TNBS-induced colitis group decreased compared with the control (sham-1, sham-2) group, while the treatment with TFA significantly rescued TNBS-induced colon damage (Figures 1C, D) and decreased the CMDI score (Figure 1E). The histopathological examination showed that in the colon tissue of the TNBS-induced group, the inflammatory cells infiltrated into the mucosa, there was loss of goblet cells, the epithelium exfoliated, and there was distortion of crypts. All these symptoms were substantially improved by TFA treatment, and the effect of TFA intervention is better in modeling than after modeling (Figure 1F).

### Total Flavone of *Abelmoschus manihot* L. Medic Treatment Protects Against 2,4,6-Trinitrobenzene Sulfonic Acid-Induced Colonic Fibrosis

**FIGURE 2:**
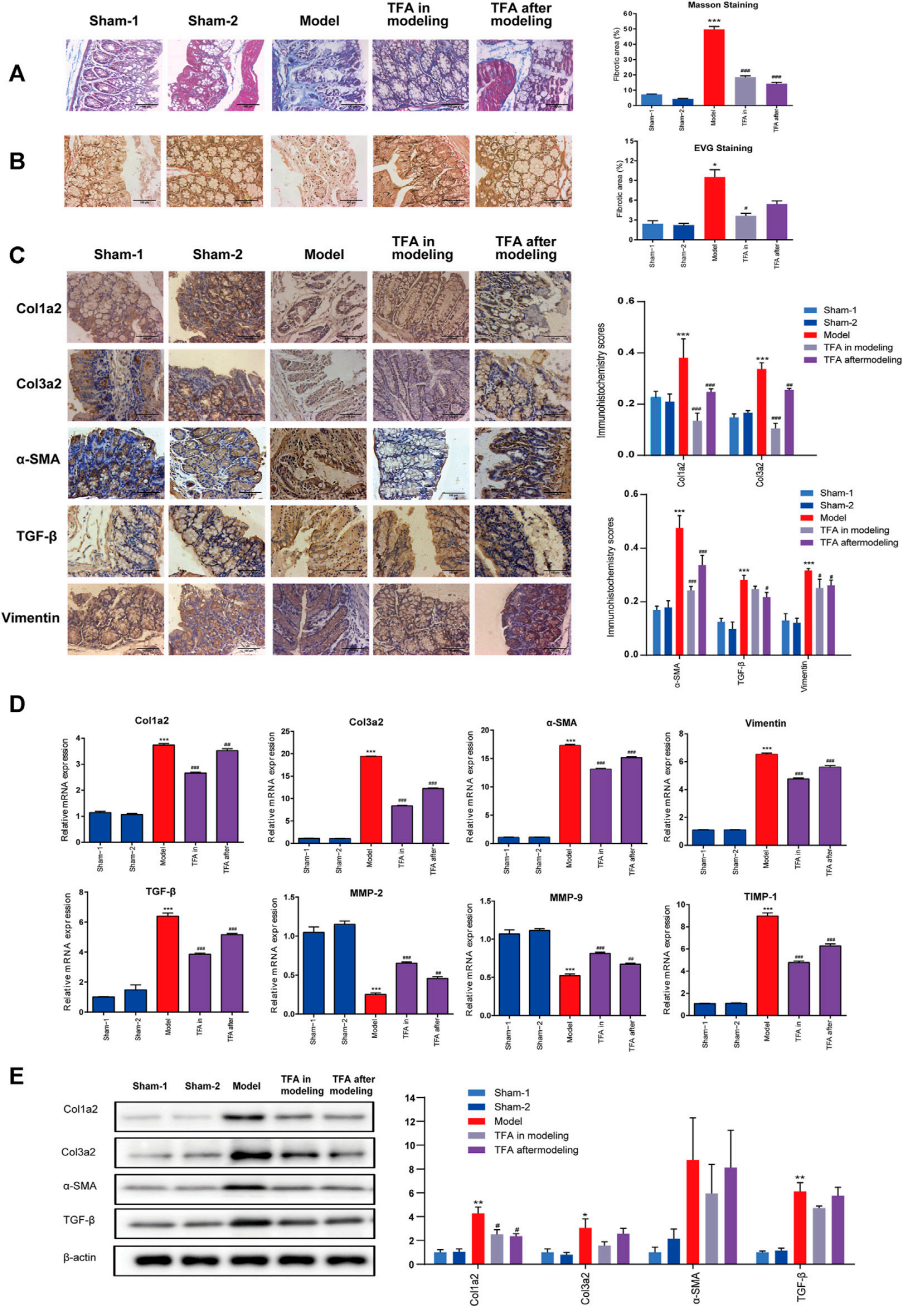
TFA inhibits fibrotic changes in the colon of TNBS-treated mice; **(A)** Masson’s trichrome staining; **(B)** Verhoeff’s von Gieson (EVG) staining assay. (×100). **(C)** Immunohistochemical staining for fibrosis-related proteins in mice from different groups. TNBS induction could significantly increase the expressions of fibrosis-related collagens (col1a2, col3a2), TGF-β, smooth muscle actin (α-SMA), and vimentin, and TFA treatment in modeling or after modeling could reverse the phenomenon. **(D)** The mRNA expression of fibrosis-related proteins was determined using quantitative real-time PCR. **(E)** The fibrosis-related protein expression was detected by Western blot analysis. Data are shown as the mean ± SEM. *p < 0.05, **p < 0.01, and ***p < 0.001compared with sham-1 and sham-2 groups, ^#^ *p* < 0.05, ^##^ *p* < 0.01, ^###^ *p* < 0.001 compared with the model group.

As evidenced by Masson staining and EVG staining, intestinal fibrosis was significantly increased in the colons of the mice after repeated TNBS administration (Figures 2A, B). In sham-1 and sham-2 groups, the blue staining of collagenous fibrous tissue was present mainly in the submucosa after Masson staining. Some narrow bands of blue staining were seen between the smooth muscle fibers of the muscularis propria. In TNBS-induced mice, the blue staining collagen in the submucosa was increased. The same result was observed by EVG staining, and collagen was stained red. Compared with the TNBS-induced group, the amount of collagen was decreased in the submucosa in the TFA in the modeling group, which confirms that TFA could intervene in the development of colon fibrosis in chronic colitis.

### Total Flavone of *Abelmoschus manihot* L. Medic Alleviated 2,4,6-Trinitrobenzene Sulfonic Acid-Induced Inflammation and Fibrosis in the Colon of Mice by Regulating the Th17/Treg Balance

**FIGURE 3:**
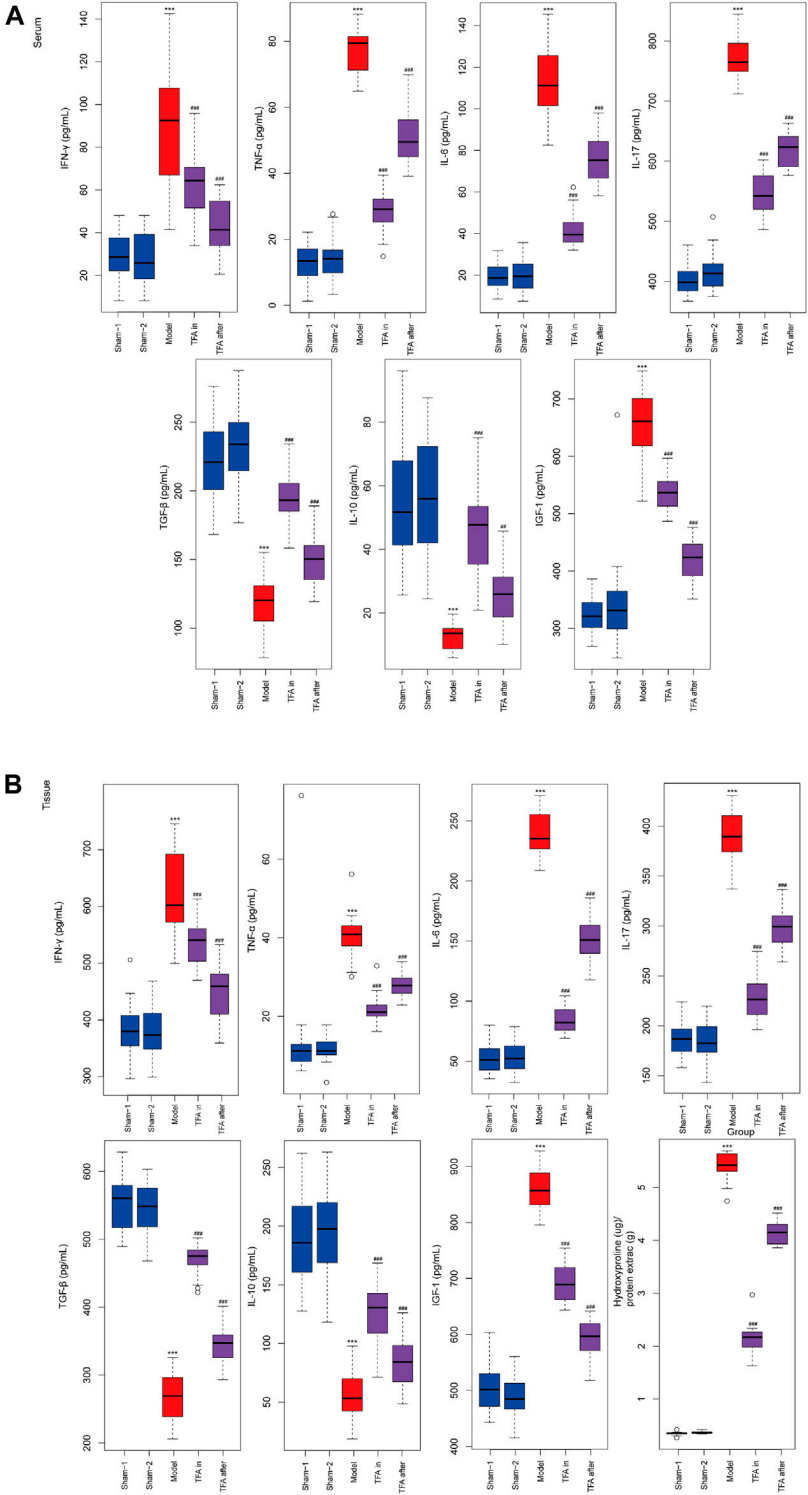
Effects of TFA on the inflammatory cytokine production in serum and tissues level of mice. TFA downregulated the expression of pro-inflammatory factor TNF-α, IFN-γ, IL-6, and IL-17 in TNBS-induced chronic colitis. Conversely, elevated protein levels of anti-inflammatory factors IL-10 and TGF-β. **(A)** ELISA for inflammatory cytokine in serum of each group. **(B)** ELISA for inflammatory cytokine in colon tissue of each group. Data are shown as the mean ± SEM. *p < 0.05, **p < 0.01 and ***p < 0.001 compared with sham-1 and sham-2 groups, ^#^ *p* < 0.05, ^##^ *p* < 0.01, ^###^ *p* < 0.001 compared with the model group.

To further explore the protection mechanism of TFA on the TNBS-induced chronic colitis model, the inflammatory factors in serum and colon tissue were detected by ELISA. There was significant upregulation of the Th17cell induced differentiation factor IL-6 and specific cytokines IL-17 both in serum and tissues after TNBS induction. Conversely, the secretion of Treg cell-specific cytokines was decreased, namely, IL-10 and TGF-β in the model group. The intervention of TFA increased the levels of IL-10 and TGF-β, and downregulated the expression of pro-inflammatory factors IL-17, IL-6, TNF-α, and IFN-γ in TNBS-induced chronic colitis (Figure 3). Taken together, TFA restores the balance of Th17/Treg cells with TNBS-induced chronic colitis to maintain intestinal immune homeostasis.

### Total Flavone of *Abelmoschus manihot* L. Medic Reduced Extracellular Matrix Deposition and Collagen Production *In Vivo* in the Colon

Proven by the above ELISA, TFA treatment significantly attenuated the increase in IGF-1 in colon tissue, which exerts a profibrotic factor (Figure 3B). Simultaneously, a decrease in hydroxyproline (HYP) expression was observed (Figure 3B). This is the main constituent of collagens whose expression is high in fibrotic tissue. We performed immunohistochemistry to evaluate the expression levels of fibrosis-related collagens (col1a2, col3a2), smooth muscle actin (α-SMA), vimentin, and TGF-β in the intestinal tract. Compared with the sham-1 and sham-2 groups, significant deposition of col1a2, col3a2, vimentin, α-SMA, and TGF-β in the colonic tissue from the TNBS group, and TFA treatment in modeling or after modeling, could reverse the phenomenon (Figure 2C).

Furthermore, a significant increase in col1a2, col3a2, α-SMA, and TGF-β was also confirmed by WB and qRT-PCR, while the expression decreased after the administration of TFA. α-SMA and vimentin are markers of the activated myofibroblast phenotype (Alfredsson and Wick, 2020) (Figures 2D, E). Increasing the expression of α-SMA and vimentin indicated that fibroblasts have differentiated into myofibroblasts and then produced a large amount of ECM. All these data indicated that TFA can inhibit fibrotic factors, reduce myofibroblast cell production as well as collagens, and prevent or reverse chronic colitis fibrosis.

Increasing evidence indicates that an unbalance between production and degradation of ECM, mediated by MMPs and TIMPs, will promote intestinal fibrosis in CD (Lakatos et al., 2012). Therefore, we tested the effect of TIMPs and MMPs in colonic tissue homogenates using PCR. As shown in Figure 2D, the expression of TIMP-1 was significantly upregulated, and MMP2/9 were significantly downregulated in the colonic tissue of mice with TNBS-induced colitis. The trend can be significantly reversed by treating with TFA in/after modeling. This proved that TFA could attenuate ECM deposition by regulating the balance between TIMPs/MMPs.

## Discussion

Intestinal fibrosis is a serious complication of IBD, which is clinically more apparent in CD. Despite significant advances in therapeutic strategies over the last decade, there is no specific and effective treatment for it. The process of intestinal fibrosis is complex and multifactorial, and single-target approaches are insufficient (Rieder and Fiocchi, 2009). Previous studies had demonstrated that TFA alleviates renal fibrosis in diabetic nephropathy and chronic kidney disease model (Chen et al., 2012; Mao et al., 2015). Increasing evidence supports the anti-inflammatory effects of TFA in IBD, through protecting the gut mucosal barrier and improving gut microbial disturbance (Zhang D. et al., 2019a; Zhang W. et al., 2019b; Wang et al., 2021). Based on the above studies, we presume that TFA may be an ideal therapeutic agent of colonic fibrosis.

**FIGURE 4:**
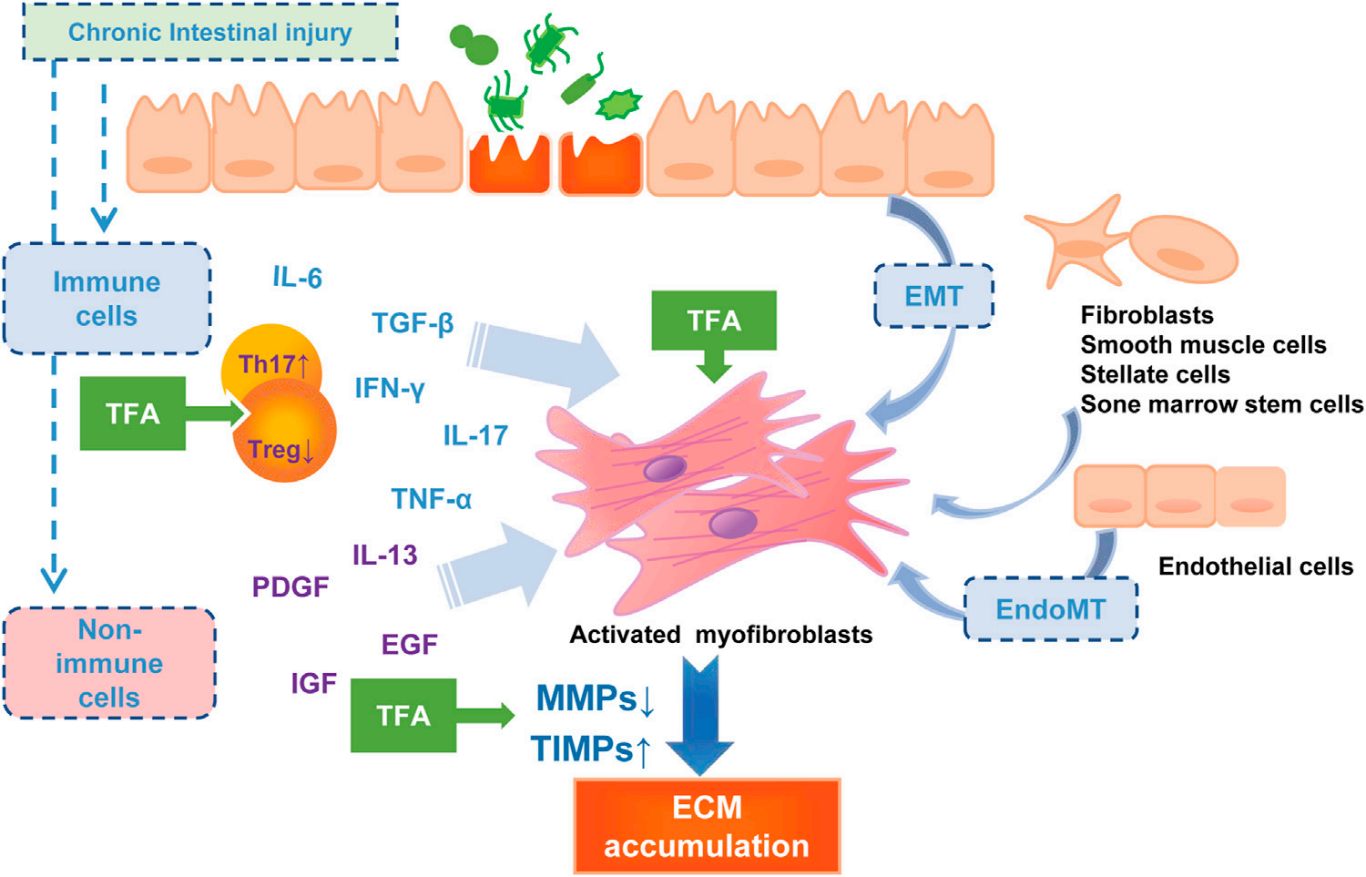
The pathophysiology of intestinal fibrosis and the possible targets of TFA intervention. PDGF, platelet-derived growth factor; IGF, insulin-like growth factors; EGF, epidermal growth factor.

In this study, we reported the therapeutic effect of TFA against experimental intestinal inflammation and fibrosis using the TNBS-induced colitis mice model. TNBS-induced colitis is a model for CD sharing similar pathophysiologic processes and significant properties with humans (Antoniou et al., 2016). Our study demonstrated that the oral administration of TFA with anti-inflammatory and anti-fibrotic activity effects significantly improved the course and macroscopic finding of TNBS-induced chronic colitis (DAI, colon weight, length, adhesions, strictures, and ulcerations), as well as the histological severity of the fibrosis of the colonic wall. The possible contribution of TFA in the anti-intestinal fibrosis in the CD model is shown in Figure 4.

Inflammation is a strong activator of fibrosis progress (Rieder et al., 2017). Induced by multiple pro-inflammatory cytokines, the mesenchymal cells increase, and collagen-rich ECM is deposited, and then the fibrosis process initiate. The Th17 cells that produce IL17 have been considered to be a new T-cell population that plays a central role in CD-associated fibrosis (Ray et al., 2014). More shreds of evidence showed that IL17 induces EMT on epithelial cells and exerts a variety of pro-inflammatory effects on fibroblasts and colonic myofibroblasts (Yagi et al., 2007; Brand, 2009; Biancheri et al., 2013; Zhang et al., 2018). Anti-IL-17A antibodies have been reported to inhibit intestinal fibrosis development through repressing EMT (Zhang et al., 2018). Additionally, IL-17A−/− reduced fibrosis in inflammatory mouse skin models (Okamoto et al., 2012). In the current study, we found that TNBS significantly elevated colonic Th17 differentiation and inhibited Treg cell differentiation, which may advocate an enhanced immune-inflammatory response. Oral administration of TFA at or after modeling significantly attenuates the production of IL-6 and IL-17 in the sera and the tissue of colitis mice, and increased the Treg cell cytokines such as IL-10 and TGF-β. Thus, we deem that TFA coordinating the regulation of Th17 and Treg would improve the inflammatory intestinal environment and, consequently, decrease intestinal fibrogenesis.

There is ample evidence that inflammation-independent mechanisms of fibrosis exist (Graham et al., 1988; Curciarello et al., 2017; Rieder et al., 2017). Intestinal fibrosis is characterized by the deposition of ECM, produced by activated intestinal mesenchymal cells, which exist in three distinct interrelated forms, myofibroblasts, fibroblasts, and smooth muscle cells (Li et al., 2019). All intestinal ECM-producing cells are synergistic and are controlled by numerous molecular mediators among which the TGF-β is the most potent mediator (Beddy et al., 2006; Curciarello et al., 2017). In the present study, the prophylactic oral administration and oral therapeutic of TFA prevented the progression of colonic fibrosis in TNBS-induced chronic colitis *via* suppression of TGF-β and, IGF and α-SMA expression to reduce mesenchymal cell production.

An imbalance due to reduced MMP activity and/or increased expression of TIMPs may lead to the excessive deposition of ECM with subsequent fibrosis in Crohn’s disease (OʼShea and Smith, 2014). This theory was confirmed by the previous finding on the specimens, where MMPs had a lower expression, while TIMP-1 is overexpressed in the mucosa overlying strictures in patients with CD (Di Sabatino et al., 2009). We used qRT-PCR analysis to test the expression of MMPs known to be regulated in CD and showed that MMP-9 and MMP-2 were upregulated, but TIMP-1 was downregulated after the TFA treatment in the TNBS-induced chronic colitis mice model. MMP-9 and MMP-2 belong to gelatinase, and they can degrade denatured collagen of all types including type IV collagen (Ravi et al., 2007). In terms of mechanism, TFA may relieve fibrosis by reverting TIMP-1-mediated inhibition of MMP2/9 activity and counteract excessive ECM deposition.

However, some differences need to be noted in this study. Goffin et al. (2016) found that inhibiting MMP-9 that was performed significantly reduces collagen deposition and intestinal fibrosis in the heterotopic transplant mouse model. It seems to suggest that MMP-9 plays a catalytic role in intestinal fibrosis. The mechanism may be related to the anti-inflammatory effect and epithelial cell adhesion inhibition (Ravi et al., 2007). The different results in MMP studies may relate to the modeling method, pleiotropic function of MMPs, and differential regulatory mechanisms between various MMP family members. The possible underlying mechanisms need to be addressed by further studies.

In conclusion, our study here explored that TFA alleviated mucosal inflammation as well as fibrosis in the colitis model. This suggests that TFA might represent a novel therapeutic strategy to inhibit the progressing or even to reverse intestinal fibrosis.

## Data Availability

The raw data supporting the conclusions of this article will be made available by the authors, without undue reservation.
